# Supplementary material for: Post-traumatic stress disorder and associated factors among internally displaced persons in Africa: A systematic review and meta-analysis
Source: PLoS One. 2024 Apr 1;19(4):e0300894. doi: 10.1371/journal.pone.0300894 (PMC10984478; doi:10.1371/journal.pone.0300894)
Supplement: S2 File — (DOCX) [file pone.0300894.s002.docx]

Results of JBI Quality Assessment for cross-sectional study

| Studies | Clear eligibility criteria | Description of study subject and  study setting | Valid and reliable method to measure the  exposure | Standard criteria used for measurement  of the condition | Identification of confounding factors | Develop of strategies to deal with confounding  factors | Valid and reliable method to measured outcomes | Appropriate statistical analysis | Total score out of 8 | Level of bias |
| --- | --- | --- | --- | --- | --- | --- | --- | --- | --- | --- |
| Asnakew et al., 2019 | Yes | Yes | Yes | Yes | N/A | Yes | Yes | Yes | 7 | Low |
| Madoro et al.,  2020 | Yes | Yes | Yes | Yes | N/A | Yes | Yes | Yes | 7 | Low |
| Makango et al., 2023 | Yes | Yes | Yes | Yes | N/A | Yes | Yes | Yes | 7 | Low |
| Masau et al.,  2018 | Yes | Yes | Yes | Yes | N/A | No | Yes | Yes | 6 | Low |
| Ali et al.,  2023 | Yes | Yes | Yes | Yes | N/A | Yes | Yes | Yes | 7 | Low |
| Elhabiby et al.,  2014 | Unclear | Yes | Yes | Yes | N/A | No | Yes | Yes | 6 | Low |
| Roberts et al., 2009 | Yes | Yes | Yes | Yes | N/A | Yes | Yes | Yes | 7 | Low |
| Roberts et al.,  2008 | Yes | Yes | Yes | Yes | N/A | Yes | Yes | Yes | 7 | Low |
| Veling et al.,  2013 | Unclear | Yes | Yes | Yes | N/A | Yes | Yes | Yes | 6 | Low |
| Sheikh et al.,  2014 | Yes | Yes | Yes | Yes | N/A | Yes | Yes | Yes | 7 | Low |
| Aluh et al., 2019 | Yes | Yes | Yes | Yes | N/A | Yes | Yes | Yes | 7 | Low |
| Ibrahim et al.,  2023 | Yes | Yes | Yes | Yes | N/A | Yes | Yes | Yes | 7 | Low |
| Faronbi et al.,  2021 | Unclear | Yes | Yes | Yes | N/A | Yes | Yes | Yes | 6 | Low |
| Nwoga et al.,2019 | Yes | Yes | Yes | Yes | N/A | Yes | Yes | Yes | 7 | Low |
