# Supplementary material for: Post-traumatic stress disorder and associated factors among internally displaced persons in Africa: A systematic review and meta-analysis
Source: PLoS One. 2024 Apr 1;19(4):e0300894. doi: 10.1371/journal.pone.0300894 (PMC10984478; doi:10.1371/journal.pone.0300894)
Supplement: Attachment — Submitted filename: Response to Reviewers.docx [file pone.0300894.s004.docx]

**Responses to the review’s comments**

**Dear PLOS ONE editorial team,**

Thank you for giving us the opportunity to submit a revised draft of the manuscript, and we would also like to thank you for your crucial comments on our paper (Manuscript ID: **PONE-D-23-40921**). We are very concerned and have combined all the suggested comments provided, which we believe strengthen our paper, and we hope this will render our paper eligible for consideration for publication in your reputed journal. We appreciate the time and effort that you and the reviewers dedicated to providing feedback on our manuscript and are grateful for the insightful comments and valuable improvements to our paper.

The authors would like to inform you that we have addressed the comments and recommendations made by both reviewers and the editor, point by point. In addition, throughout our revision, we made our best corrections too. All changes made to the original version are highlighted using tracking changes and attached as “**Revised Manuscript with Track Changes**." The unmarked copy of the manuscript is also attached as “**Manuscrip**t.” In addition, please see below a rebuttal letter that responds to each point raised by the academic editor and reviewers, and this letter is also attached to the submission as “**Response to Reviewers**."

**Response to editor’s comments**

1…Please ensure that your manuscript meets PLOS ONE's style requirements, including those for file naming. The PLOS ONE style templates can be found at

https://journals.plos.org/plosone/s/file?id=wjVg/PLOSOne_formatting_sample_main_body.pdf and

https://journals.plos.org/plosone/s/file?id=ba62/PLOSOne_formatting_sample_title_authors_affiliations.pdf

**Author response:** Dear the academic editor, we are grateful for your comments regarding the tool used to measure satisfaction. The tool is validated and have been used in Ethiopian population, we have also examined the reliability and found in the acceptable range.

2…We noticed you have some minor occurrence of overlapping text with the following previous publication(s), which needs to be addressed:

https://doi.org/10.1371/journal.pone.0287996

In your revision ensure you cite all your sources (including your own works), and quote or rephrase any duplicated text outside the methods section. Further consideration is dependent on these concerns being addressed.

**Author response:** Dear the academic editor, we are grateful for your comments regarding the tool used to measure satisfaction. The tool is validated and have been used in Ethiopian population, we have also examined the reliability and found in the acceptable range.

Dear Academic Editor, we have now cited and rephrased the duplicated text as appropriate. Each suggested comment has been reviewed, edited and modified accordingly. All the authors are grateful for all your comments, as they have all helped to improve our revised manuscript for publication.

3…Additional Editor Comments:

Kindly address all reviewers' recommendations.

**Author response:** Dear the academic editor, we are grateful for your comments regarding the tool used to measure satisfaction. The tool is validated and have been used in Ethiopian population, we have also examined the reliability and found in the acceptable range.

**Response to reviewers’ comments**

**Reviewer #1**: General

Thank you, authors, for coming with this interesting topic. I think, the finding from the current study will be very important for the regional policy makers. in order to further improve the manuscript, authors need to consider some comments listed below. Initially, authors are suggested to go through their document and edit it for some spelling error and repetition of sentences comprising the same message/idea. Furthermore, Authors are also advised to use Abbreviation consistently in the document once decided to do so.

**Author response:** Dear reviewer, we are very grateful for your general comments and suggestions that we believe it could improve the quality of this paper. We have also shared your comments and concerns, taking your valuable comments and suggestion, we have corrected and made the necessary changes accordingly. We hope you found it improve.

**Abstract**

**Method**: sentence number 1 and 2 have similar message/idea, so authors are advised to merge them in one sentence.

**Author response:** Dear reviewer, thanks for your valuable comments and suggestions to merge these sentences, and we shared your comments and revised it accordingly.

Result: 4-7, 8-11 and 12-16 traumatic events were significantly associated with PTSD. However it is not clear weather this number indicates number of traumatic events experienced or frequency of exposure to traumatic events, hence, the two were conceptually different. e.g an individual may be exposed to various traumatic events at single occasion or may experience repeated traumatic events on different occasions. Moreover, the three category (4-7,8-11,12-16) were reported to be associated with PTSD, unless you are interested in examining which category is highly significant, you can merge them as ≥ 4 traumatic events.

**Author response:** Thanks for your comments and we have shared your comments and suggestions in this regard. However, this number indicates the number of cumulative traumatic events experienced by IDPs whether at a single occasion or on different occasions. We also revised and tried to clarify on it accordingly. Regarding category, as you mentioned that we aimed to appreciate the significance of different category of cumulative number of traumatic events because difference in exposure of traumatic events may have an impact on the occurrence of PTSD.

Introduction

Line 63 “PTSD is a serious” the word serious is a vague word, please be advised to replace it with other appropriate word.

**Author response:** Thank you and we have revised it accordingly.

Line 74 “insufficient mental health professional” better replaced with “insufficient mental health services” because the latter one can best address both facility and professionals.

**Author response:** Dear reviewer, thank you for your important suggestion and we have revised it accordingly.

Line 92-95: too long sentence and difficult to grasp its meaning. Please rewrite it.

**Author response:** Dear reviewer, thank you very much and corrected accordingly.

Line 102-103: “a comprehensive study that can address the overall public health impact of IDPs in terms of causing PTSD…” please replace IDPs with internal displacement, otherwise it gives meaning other than the intended one.

**Author response:** Thank you for your valuable corrections, and we have revised it accordingly.

**Method**

Line 122-123: the sentence begin with “the following database”… lacks to give full meaning please revise it.

**Author response:** Dear reviewer, we are very grateful for your insightful comments, and we have revised it accordingly.

Line 123-125: please delete “the search strategy aimed to identify studies reporting on PTSD prevalence rates and associated factors among internally displaced persons (IDPs) in Africa”.

Inclusion criteria: I suggest you to merge and write it as paragraph, not listed bullet

**Author response:** Thanks, we shred your comments, and deleted accordingly.

Line 148: please delete “in this study research articles like”

**Author response:** Thanks, and we have corrected it accordingly.

Line 176-177: “it was evaluated using the JBI” repetition of idea in prior sentence so delete it.

**Author response:** Thanks, and we have corrected it accordingly.

Line 183: please delete “there are two main outcomes for this review” because the readers can easily understand this from the title

**Author response:** Thanks, and we have corrected it accordingly.

191: The I2 score range used to categorize the degree of heterogeneity overlap each other, and I think this is not correct. Authors are suggested to revise it. May be you can refer to <https://doi.org/10.1007/s13312-022-2500-y>

**Author response:** Dear reviewer, thank you for your critical comment. All authors have accepted it and corrected accordingly.

**Result**

Please revise your result for consistency, logical flow of ideas and typos. There is unnecessary detail throughout the result section particularly under factors associated with PTSD. There is detailed information both in text narration and tables, thus I suggest authors to minimize duplication of information.

**Author response:** Dear reviewer, we shared your concerns and taking your valuable suggestions, we have revised it accordingly and we hope you have found it improved.

Result presented in the same style e.g. “unemployed were 1.92 times more likely to have PTSD than employed IDPs... “, this style appeared to be commonly used throughout the document. In order to make the document more attractive and help the readers to make sense of it, it is crucial to present the result in different, clear and intelligent fashion. I recommend authors to go through the document and make some amendments with regard to this point.

**Author response:** Dear reviewer thank you for your comments and we have revised it accordingly.

On quality appraisal of the included studies, I think the authors need to clearly describe full result (each assessment question with corresponding given score) to ensure that detailed objective scoring are disclosed beforehand and will be reproduced.

**Author response:** Thank you for your comment and suggestion. Following your comment, we have now clearly described the full result of the quality assessment of the included studies (each assessment question with the corresponding score) and attached it as a supplementary file 2 (**S2 Table**).

Line 213-214: “the included articles were conducted b/n 2008-2023” please replace “conducted” with published. Because you don’t know when the studies were carried out but when the article is published.

**Author response:** Thanks, and we have corrected it.

Line 220 - Please delete “were used to determine the pooled prevalence of PTSD among IDPs.”

**Author response:** Thanks, and we have corrected it accordingly.

Line 228-229 - the sentence has to be stated in method section.

**Author response:** Thanks, and we have moved it to the method section.

Line 259 there is unnecessary detail, I suggest the authors to directly state finding from the included studies, please delete “Based on the results of the 14 included studies, the analysis showed that”

**Author response:** Thanks, and we have corrected it accordingly.

Line 261 please revise punctuation error

**Author response:** Thanks, and we have corrected it accordingly.

Line 263: please delete “being exposed to”, and elsewhere in the document please replace more likely to “develop” with appropriate word, because with pooled prevalence result from cross sectional studies it is difficult to talk about incidence (“to develop” is in favor of new incidence)

**Author response:** Thanks, and we have corrected it accordingly.

Line 269 and elsewhere in documents please carefully revise spelling error eg. Metal analysis to meta-analysis.

**Author response:** Thanks, and we have tried to correct potential spelling errors accordingly**.**

**Discussion**

Authors have tried to explain the possible justification toward the discrepancy between this study and other study finding. However, it is not clear whether the stated justification is to higher or lower prevalence rate, also it is not clearly stated on how those factors may account to the observed difference. Moreover, only one reference is cited to indicate evidence that supports their long justification, which is inappropriate as to me.

**Author response:** Dear thanks for your valuable comments and we have presented the justification to a lower and a higher prevalence rate independently, and included potential evidences to justify the implications.

Line 317-321 almost repetition of result, please delete it

**Author response:** Thanks, and we have deleted.

Line 344: “exposed to” better replaced by ‘associated with’

**Author response:** Thanks, and corrected.

Line 358-359 please delete it because it is repetition “Employment status can significantly impact an individual's risk of developing PTSD among IDPs”.

**Author response:** Thanks, and corrected.

Strength and limitation

Line 439: “The study's strengths outweigh its limitations”. I suggest authors to revise/delete this sentence, as to me it is not reasonable to claim this at this time.

**Author response:** Thanks, and corrected it accordingly.

**Reviewer #2:**

This is an interesting paper and the content is very good but I suggest the paper can be improved in the following ways:

**Abstract**

Please modify the headlines based on the format of the journal

**Author response:** Dear the reviewer, we are grateful for your general comments and suggestions that we believe it could improve the quality of the paper.

Regarding the headlines of the abstracts, we have shared your comments and suggestions, and we revised and corrected it accordingly.

-In the method section, please include the year of the study, sampling method, data analysis method.

**Author response:** Thank you for your helpful comments. The comment has been accepted and corrected accordingly.

**Introduction**

In this section, please bring this item

1- Definition of the research problem

2- The magnitude and importance of the study variable

3- Bringing the variable epidemiology information of the study at the world level, the country of India and

the place of study

4- Bringing theoretical knowledge about the importance of studying

5- Expressing the necessity of conducting the study

Finally, the practical purpose of the study should be stated

**Author response:** Dear the reviewer, we are grateful for your general comments and suggestions that we believe it could improve the quality of the paper.

We have shared your comments and concerns, taking your valuable comments and suggestion, we have corrected and made the necessary changes accordingly.

**Discussion**

In the discussion section, it is necessary to compare the main results of the study with the results of other studies in this field,

To strengthen the article, especially in the introduction and discussion section the following studies are suggested, please to use and cited and add to article reference

- Post-traumatic stress disorder in medical workers involved in earthquake response: A systematic review and meta-analysis

- Anxiety, stress and depression levels among nurses of educational hospitals in Iran: Time of performing nursing care for suspected and confirmed COVID-19 patients

- Prevalence of workplace violence against health care workers in hospital and pre-hospital settings: An umbrella review of meta-analyses

**Author response:** Thank you for your thoughtful comments. We have now modified, revised and corrected the discussion of our results. All the authors would like to thank you for all your comments, which helped to improve the manuscript for publication. We hope that you are now interested in our revised manuscript.

**Conclusion**

− What are your suggestions for future studies?

**Author response:** Thanks, and included in the discussion section.
