# Supplementary material for: Post-traumatic stress disorder and associated factors among internally displaced persons in Africa: A systematic review and meta-analysis
Source: PLoS One. 2024 Apr 1;19(4):e0300894. doi: 10.1371/journal.pone.0300894 (PMC10984478; doi:10.1371/journal.pone.0300894)
Supplement: Attachment — Submitted filename: 'Response to Reviewers'.docx [file pone.0300894.s005.docx]

**Responses to the review’s comments**

**Dear PLOS ONE editorial team,**

Thank you for giving us the opportunity to submit a revised draft of the manuscript, and we would also like to thank you for your crucial comments on our paper (Manuscript ID: **PONE-D-23-40921R1**). We are very concerned and have combined all the suggested comments provided, which we believe strengthen our paper, and we hope this will render our paper eligible for consideration for publication in your reputed journal. We appreciate the time and effort that you and the reviewers dedicated to providing feedback on our manuscript and are grateful for the insightful comments and valuable improvements to our paper for publication.

The authors would like to inform you that we have addressed the comments and recommendations of the handling editor point by point. In addition, throughout our revision, we made our best corrections too. All changes made to the original version are highlighted using tracking changes and attached as **&#39;Revised Manuscript with Track Changes&#39;**. The unmarked copy of the manuscript is also attached as **&#39;Manuscript&#39;**. In addition, please see below a rebuttal letter that responds to each point raised by the handling editor, and this letter is also attached to the submission as **&#39;Response to Reviewers&#39;**.

**Response to editor’s comments**

**Comments from the handling editor:**

1. PTSD and PTS symptoms are two different things, and I understand that there can be strong limitations in the field to assess actual PTSD, and most of times only screenings can be done.

In order to avoid this potential bias in the global estimates, I recommend including a table with information regarding the tool used by the original studies for assessing PTSD. In this sense, did the original studies assess PTSD with clinical confirmation? Or did they assess screening of PTS symptoms without confirmation? Or mix of both assessments?

**Author’s response:** Dear Editor, thank you very for your recommendation to include a table providing information about the assessment tools used in the original studies This is an excellent suggestion to address potential biases and provide transparency regarding the assessment methods employed in the included studies.

By including a table with this information, readers can have a clear understanding of how PTSD was assessed in each study and whether clinical confirmation or screening of PTS symptoms was utilized. This can help in evaluating the quality of evidence and the potential impact of different assessment approaches on the prevalence estimates.

To address your specific question, we have now included a table that clearly shows details such as the name of the assessment tool used and the methods used to collect PTSD data, whether it was clinical confirmation or screening, or a combination of both. This will provide a comprehensive overview of the assessment methods employed in the original studies and allow for a more nuanced interpretation of the findings.

Including this table will enhance the transparency and robustness of our systematic review and meta-analysis, providing valuable information to the readers and enabling a more accurate assessment of the pooled estimate PTSD prevalence. Furthermore, it will help in identifying potential sources of heterogeneity and exploring the impact of different assessment strategies on the overall findings. ***Reference: See our revised manuscript Table 2.***

Thank you again for your valuable suggestion.

2. I also recommend a subgroup MA including clinically confirmed cases and positive screening cases as different sub-groups.

**Author response:** Dear editor, thank you for your valuable feedback. We agree that including clinically confirmed cases and positive screening cases as different sub-groups in our subgroup meta-analysis of participants with PTSD would provide a more comprehensive understanding of the condition. We have carefully considered your suggestion and have made the necessary revisions to our analysis.

We have conducted subgroup analysis and computed the pooled prevalence of PTSD using clinically confirmed cases and positive screening cases separately, and we have clearly explained their results in the discussion section. We have included forest plots to visually present the results and have clearly addressed potential limitations or biases in the discussion section. We believe that these additions have strengthened our analysis and provided more meaningful insights into PTSD among different sub-groups of participants. ***Reference****:* ***See our revised manuscript Figure 4 and Figure 5, also see discussion section, paragraph 3.***

Thank you for your valuable input. We sincerely appreciate your valuable feedback and the opportunity to improve our manuscript for publication.

We are very grateful to the editor and the two reviewers for their time and effort in improving our manuscript. We thank them for their scientific comments.
